# Individualized management of giant anterior meningoceles-case series

**DOI:** 10.1097/MD.0000000000019631

**Published:** 2020-04-03

**Authors:** YueLong Wang, Bin He, Jinhao Yang, Jianguo Xu, Jiagang Liu, Siqing Huang

**Affiliations:** a Department of Neurosurgery, West China Hospital; b Department of Obstetrics, West China Second Hospital, Sichuan University, Chengdu, PR China.

**Keywords:** anterior myelomeningocele, congenital, individualized management

## Abstract

**Rationale:**

Anterior spinal meningoceles are rare neuroanatomic abnormality formed by protrusion of the spinal meninges through a defect in the vertebral column. Presently, therapeutic options for anterior spinal meningoceles are still controversial. The objective of this study is to discuss the individualized management of giant anterior spinal meningoceles.

**Patient concerns and Diagnoses:**

We analyzed 4 patients with anterior spinal meningoceles between 2007 and 2014 in our department by retrospective chart review, two of whom were anterior sacral meningoceles (ASMs), and another2 were intrathoracic meningoceles (ITMs).

**Interventions and outcomes:**

Patients mainly presented with compressive symptoms including rectal irritation, dyspnea (patient 3) and fixed neurologic deficits (patient 4). Three out of 4 patients received surgical treatment, one of which underwent reoperation. After surgery, meningoceles in 1 patient completely disappeared. Two patients acquired the stability of the size of the meningoceles.

**Lessons:**

Management of anterior spinal meningoceles often requires precise treatment based on the different conditions of each patient. Surgical intervention has been proposed for the treatment of symptomatic anterior spinal meningoceles. The goal of surgery is to safely disconnect the linkage between the cyst and CSF from subarachnoid space to prevent further enlargement of the cyst or reaccumulating of cystic fluid.

## 1 Introduction

Anterior spinal meningoceles are very rare. They are formed by protrusion of the spinal meninges through defects in the vertebral column.^[1]^ This disease most commonly results from dystrophic abnormality at the sacral level but can occur higher up, such as cervical and thoracic levels.^[2,3]^

Anterior sacral meningoceles (ASMs) were first described by Bryant in 1837.^[4]^ Considered to be congenital, ASM is the result of dural ectasia due to subarachnoid space enlargement by cerebrospinal fluid (CSF) pulsastion.^[5]^ However, the exact etiology of ASM remains unclear. ASMs, as presacral masses, can also be the manifestation of Currarino triad, which involves anorectal, sacral and presacral anomalies. In addition, they may associate with underlying connective tissue disorders like neurofibromatosis type 1 (NF-1), Marfan syndrome, and Ehlers-Danlos syndrome.^[6–8]^

Different from posterior meningoceles, ASMs usually occur in young adults. A female predominance has been reported, but not confirmed.^[9]^ They are often found incidentally as a smooth cystic mass during a rectal or pelvic examination. Most patients present with either compressive urogenital and colorectal symptoms or neurologic symptoms. In contrast to posterior defects, ASMs are not usually complicated by hydrocephalus and Chiari malformations.^[9]^

Intrathoracic meningoceles (ITM) are even rarer than ASMs, which were first described by Phol as a cystic formation of the posterior mediastinum in 1933.^[10]^ They originated from a saccular protrusion of the meninges through the pathologically dilated intervertebral foramen or a bone defect in thoracic vertebrae in the thoracic cavity. As reported, ITMs are commonly associated with NF-1.^[11]^ NF-1, also known as von Recklinghausen's disease, is an autosomal-dominant disease caused by genetic mutations of the NF-1 gene located on chromosome 17q11.2.^[12]^ According to literature, about 69% of thoracic meningoceles are associated with NF-1.^[13]^

Spinal deformities are common in patients with thoracic spinal meningoceles. The incidence of spinal deformities in NF-1 ranges from 10% to 60%, with scoliosis being the most common spinal manifestation.^[14]^ Meningoceles can be asymptomatic or present with symptoms related to compression of nerves or the spinal cord.^[12]^ Additionally, compression of the pleural cavity and other mediastinal structures can lead to symptoms like cough, dyspnea and palpitations.^[15]^ Besides, spontaneous rupture is another notable complication for patients.^[13]^ The treatment including surgical removing the large mass and restoring the spinal deformities through a thoracotomy or laminectomy.^[12]^

In this study, we described four patients with anterior spinal meningoceles (2 ASM and 2 ITM) and associated neuroanatomical malformations. And we also discussed the indications and cautions for neurosurgical treatment.

## 2 Patients and methods

Clinical data of 4 patients with anterior spinal meningoceles (2 ASMs and 2 ITMs) presenting to West China Hospital of Sichuan University during the period of 2007 to 2014, were collected. Spinal malformations were evaluated via magnetic resonance imaging (MRI). The presenting symptoms are described in Table 1. The neurosurgical considerations and sequela are described below. Written informed consent was obtained from all patients.

**Table 1:**
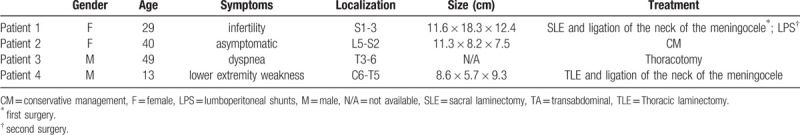
Summary of the present four cases.

## 3 Patients

### 3.1 Patient 1

**Figure 1:**
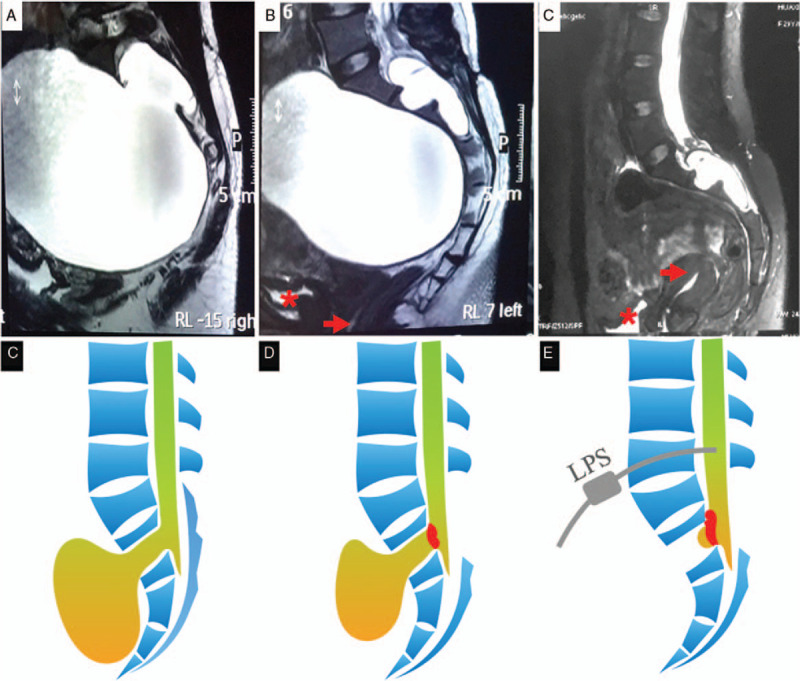
Anterior sacral meningocele in patient 1. Preoperative sagittal T2-weighted MRI (A, B) shows a large, well-defined cystic mass occupying nearly entire pelvis, compression of the uterus (B, red arrow) and bladder (B, red asterisk). After the second operation, sagittal T2-weighted MRI (C) shows complete disappearance of the meningocele, and decompression of the bladder (C, red asterisk) and uterus (C, red arrow). The schematic diagram illustrates the position of meningocele (D), the first surgical method (E), and the second surgical method (F).

A 29-year-old woman presented with infertility (Table 1). The results of the magnetic resonance imaging (MRI) at a local hospital revealed a large cystic abdominal mass consistent with an anterior sacral meningocele. The patient was sent to our center for further treatment. She denied abdominal discomfort, bowel or bladder symptoms, frequent headaches, nausea, vomiting, or visual changes. On physical examination, a large, firm, nontender mass can be palpated at her lower abdomen. Cranial nerve, somatic motor and sensory examinations showed to be normal. Unremarkable were the laboratory test results found to be. MRI revealed a large, non-enhanced, cystic abdominal mass measuring about 11.6 × 18.3 × 12.4 cm, spanning across S1 segment and S3 (Fig. 1A,B). The neck of the cyst seemed to be in continuity with the lumbar cistern, and a 7-cm defect was noted in the right posterolateral part of the S2 vertebral body. The patient received her first operation via a dorsal trans-sacral approach. After draining the cystic fluid, the neck of the meningocele opening was repaired with muscle and absorbable sutures (Fig. 1E). Her symptoms relieved significantly after the operation. However, her repeated MRI revealed an abdominal mass again after 3 months. So she received another operation via the same surgical approach as the first time. We drained the cystic fluid and reinforced the neck of the meningocele opening with muscle and absorbable sutures. After that, lumboperitoneal shunts (LPS) was performed (Fig. 1F). The patient acquired rapid postoperative recovery and discharge 5 days after the operation. Follow-up MRI was performed 1 year after surgery showing no reaccumulation of cyst fluid (Fig. 1C). And she gave birth to a baby 2 years after the operation.

### 3.2 Patient 2

**Figure 2:**
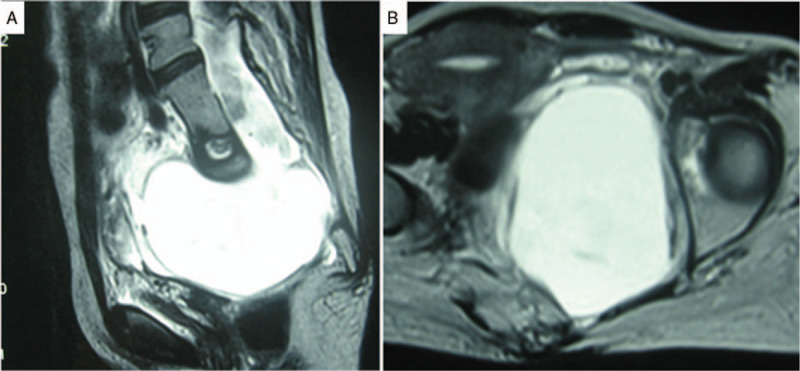
Anterior sacral meningocele in patient 2. Sagittal (A) and axial T2-weighted MRI (B) shows a large, well-defined cystic mass.

A 63-year old female was referred to our outpatient department due to pelvic cystic mass, which found by health examination (Table 1). There were no focal neurological deficits. Contrast-enhanced pelvic MRI demonstrated a large, well-defined, presacral cyst extending through defects in the anterior aspect of sacrum, without contrast enhancement (Fig. 2). The cystic fluid was similar to cerebrospinal fluid (CSF) signal intensity. There were neither nerve roots nor solid components within the mass behind uterus and rectum. A diagnosis of ASM was established based on these findings. The patient was informed about surgical management. However, she rejected surgery as a result of her current asymptomatic condition. The possible complications such as fistula, meningitis, rupture and the need for regular follow-up were told before her discharge.

### 3.3 Patient 3

**Figure 3:**
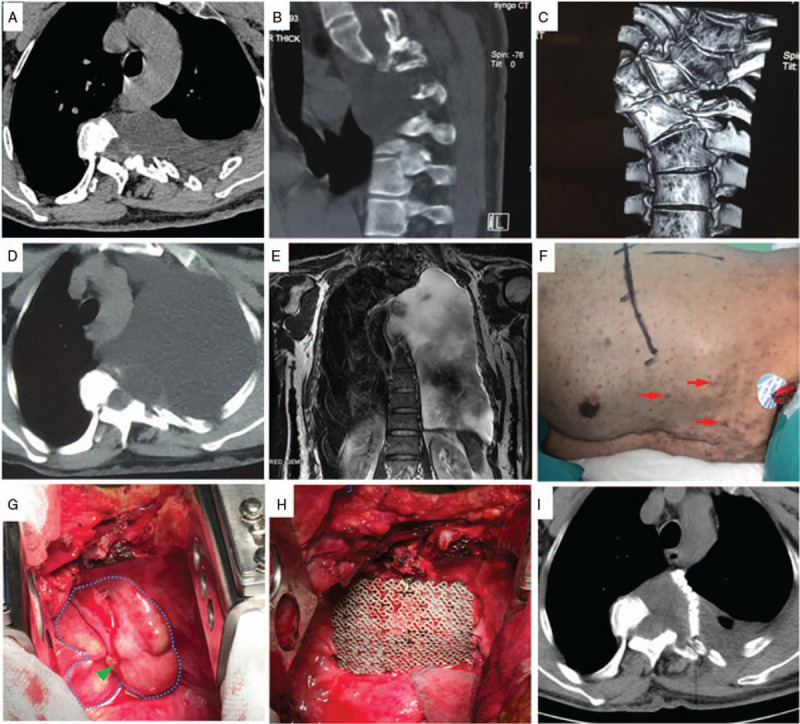
Intrathoracic meningocele in patient 3. Chest computed tomography (A) and thoracic spine three-dimensional computed tomography (B, C) shows a homogeneous cystic mass with a thin wall in the left paraspinal space with scoliosis before rupture of the cystic. Chest computed tomography (D) and coronal chest MRI show a large number of pleural effusion after the rupture of the cystic. Disseminated cafe ´-au-lait spots and subcutaneous nodules (red arrow) on the chest and abdomen of the patient (F). Intraoperative image (G) shows a meningocele with a rupture of the cystic wall (green arrowhead). After repaired the cystic wall, a piece of titanium mesh was applied to prevent meningocele expansion (H). Postoperative chest CT (I) show a small residual pseudo meningocele and mesh repair in place with little pleural effusion.

A 49-year old male complained of progressively worsening dyspnea for the previous 2 months and referred to our department for further evaluation (Table 1). He had been diagnosed with NF-1 and intrathoracic meningoceles (Fig. 3A–C) 6 years ago and has been taking conservative treatments. The aggravation of his condition was caused by cyst rupture recently (Fig. 3D, E). There was no history of infection, trauma, or spinal surgery. On physical examination, diminished breath sounds were noted on the left. In addition, there were widespread café-au-lait macules along with subcutaneous nodules (Fig. 3F). On neurological examination, no focal deficits were appreciated. Three-dimensional computed tomography and MRI of the lungs revealed the left thoracic cavity was full of fluid, and the lung tissue was not visible with severe spinal deformity (Fig. 3D, E). The left neural foramina of the fourth and fifth thoracic vertebrae were dilated, likely due to protrusion of the meninges via these foramina. A rupture of postero-lateral oriented intrathoracic meningocele was thus diagnosed. Due to the massive pleural effusion, a left thoracotomy combined with meningocele repair was performed with the assistance of a thoracic surgeon for this patient. Endotracheal intubation was achieved with a double-lumen tube to allow for single-lung ventilation when repairing the meningocele. After the release of chest water, meningocele could be seen at the posterior mediastinum with two ruptured sites on its surface (Fig. 3G). Meningocele was repaired by absorbable sutures and further strengthened by a piece of titanium mesh using a cranial plating system for the prevention of meningocele expansion (Fig. 3H). The patient recovered well, and his postoperative pulmonary function tests showed normal. Imaging of two weeks post-operation showed a complete expansion of the lung without hydropneumothorax and stability of the size of the meningocele (Fig. 3I).

### 3.4 Patient 4

**Figure 4:**
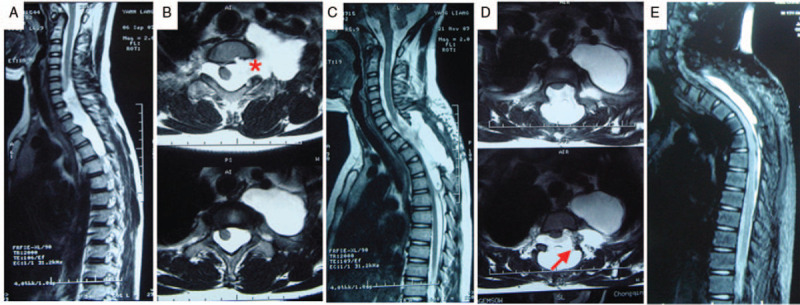
Intrathoracic meningocele in patient 4. Preoperative sagittal (A) T2-weighted MRI showing an intradural arachnoid cyst lying between C3 and T5. Axial (B) T2-weighted MRI showing a large meningocele and its connection with the arachnoid cyst at the level of T2 (red asterisk). ostoperative T2-weighted MRI (C, D) showing intrathoracic meningocele still exist with new epidural effusion. Red arrow showing the repaired site. One year after surgery, T2-weighted MRI showing shrinking of epidural effusion with a little kyphosis (E).

A 13-year-old male patient was referred to a local hospital with a history of lower extremity weakness as well as slight limb numbness. In his neurological examination, paraparesis was confirmed. The muscle strength of both legs was MRC grade 4/5 proximally and distally. The sense of pain was decreased bilaterally below the level of C7. Bilateral deep tendon hyperreflexia and positive Babinski sign were also demonstrated. Thoracic vertebra MRI revealed a cystic enlargement of the spine from C6 to T5 (Fig. 4A). This enlargement was interpreted as an intradural arachnoid cyst. One intrathoracic meningocele pouches were also found in axial MRI sections across the level from C6 to T3 with enlargement of left C7/T1 neural foramen (Fig. 4B). T1-T3 laminectomy and subsequent fastening and strengthening of the meningocele neck with muscle were performed by a local neurosurgeon. However, postoperative MRI study of the thoracolumbar spine showed intrathoracic meningocele still exists with newly-emerged epidural effusion (Fig. 4C, D), His sensorimotor dysfunction still not returned to normal. Thus, he went to our department for further evaluation. We considered that his sensorimotor dysfunction was mild, which could take conservative treatment modalities with regular follow-up. One year after surgery, his neurological function remained stable. MRI showed shrinkage of epidural effusion with a little kyphosis (Fig. 4E).

## 4 Discussion

The pathogenesis of anterior spinal meningoceles can be partially interpreted by the hypothesis of congenital neural tube defects arising during embryonic development at the stage of neurulation. Meningocele development is explained by CSF pulsation eroding the weakened spinal wall or intervertebral foramen and leading to out-pouching. However, the pathogenesis of these meningoceles is still uncertain. As patient 3 in our study, the association between ITMs and NF1 has been reported in numerous literatures.^[12,13]^ About 69% of intrathoracic meningoceles occurred in association with NF1, while only 22.4% occurred to be idiopathic without a definite cause. ASMs could be acquired as a part of syndromes such as NF1, Marfan, Ehlor-Dahlos, Currarino syndrome and may be congenital.^[16]^ ASMs are often accompanied with anorectal malformations and other presacral tumors such as lipomas, dermoid, teratoma, or epidermoid.^[17]^ Congenital ASMs may be inherited in an autosomal or sex-linked dominant manner.^[17]^

The presentation of meningoceles is highly variable. Small or late-onset isolated meningocele is usually asymptomatic. Early phase symptoms are mostly related to surrounding organs affected by mass effect. The patient may have compressive symptoms such as rectal irritation, dyspnea (patient 3), or urinary frequency or neurologic symptoms such as fixed neurologic deficits (patient 4) or uterus and bladder involvement (patient 1).^[11,16]^ In extreme cases, ASMs may present with bacterial meningitis, presumably due to a communication between the CSF and an enteric structure.^[18]^ Finally, death is the most serious consequences of meningoceles, since rare cases of fatal spontaneous rupture have been reported during labor,^[19]^ such as patient 3 in our study.

As a safe, rapid and noninvasive imaging technique with multiplanar imaging capability, MRI is the gold standard for the diagnosis of ASM. Accurate information on the shape, size, anatomical relations with surrounding organs, and intrinsic characteristics of the cystic mass may be obtained with routine MRI without the introduction of contrast agents into the spinal canal. Myelography may be useful in those cases with MRI-undetectable narrow communications.^[20]^ CT is useful to display bony anomalies and erosions and CSF density within the cyst.

Management of anterior spinal meningoceles often requires precise treatment based on the different conditions of each patient. Surgical and observational management has been both reported in the literature.^[1,11,17]^ It has been acknowledged that conservative management is an appropriate option for small, uncomplicated meningoceles (such as the patient 2), without associated tumors or pregnant patients. However, spontaneous regression of meningoceles has not been observed and life-threatening complications such as fistula, meningitis, and rupture have been described. The existence of such complications urges us to balance risk and benefit when choosing treatment modalities between conservative therapies or surgery. Regular follow-up and reexaminations should be recommended to patients undergoing conservative treatment.

In the present 4 cases, 3 of them received surgical treatment, 2 of them suffered reoperation. Various surgical approaches have been proposed for the treatment of symptomatic meningoceles, but decisions are made on a case-by-case basis. The goal of surgery should be disconnection of linkage between the cyst and CSF from subarachnoid space to prevent further enlargement of the cyst and reaccumulating of cystic fluid, with insurance of safety.^[5]^

When meningoceles occur in the sacrococcygeal region, the most commonly used surgical methods are anterior transabdominal approach and posterior approach through sacrococcygeal laminae.^[1]^ There have also been reports on the placement of cyst peritoneal and lumboperitoneal shunts using fixed pressure and programmable valves.^[21]^ However, a combination of two or more surgical approaches should be applied, as in the situation of patient 1. This patient received LPS due to a recurrence of the cyst after the first operation. LPS could reduce the CSF pressure exerting on the newly repaired neck of meningocele. And when the neck of meningocele tightly adhered together, the cyst will not appear again.

Sometimes it is easy to find the right method based on the patient's specific situation. We reported another case of ASM before, which received a posterior approach through sacrococcygeal laminae in our department after the first failure of the transabdominal approach.^[22]^ We considered that communication between the cyst and the subarachnoid space is under the fifth sacral vertebrae without nerve involvement. Thus, we took sacral laminectomy, found and ligated the neck between the cyst and the subarachnoid space. After that, the cyst in the pelvic cavity disappeared and the patient's symptoms were completely relieved.

When meningoceles occur in the thoracic region, patients often accompanied by spinal deformities, and the management will be complicated. Paramita et al reported a case of a 43-year-old woman with intrathoracic meningocele associated with NF1 and in whom shunting of the pseudo meningocele failed.^[11]^ Subsequently, a posterolateral thoracotomy was performed. The dura mater was reconstructed and primarily closed. On this closure, a Gore-Tex soft-tissue patch was placed along with polypropylene mesh and Evicel fibrin sealant, strengthened by titanium mesh. Similar to the Paramit's report, our patient 4, who experienced the rupture of intrathoracic meningocele, received left thoracotomy along with repairment of the meningocele which was performed under the assistance of a thoracic surgeon. As for large meningoceles, thoracotomy is preferred, and watertight closure of the dura in these cases is important. There have been reports of closures that have been reinforced with cyanoacrylate cement, muscle, or fascia.^[11]^ As demonstrated in our patient, titanium mesh may be another option for reinforcing the closure.

When intrathoracic meningoceles occur with a spinal intradural arachnoid cyst, and the patient's neurologic deficits were secondary to the spinal intradural arachnoid cyst not related to the intrathoracic meningocele, it is more suitable for the thoracic vertebral lamina approach, such as patient 4. However, this patient acquired poor surgical results. Communication between the cyst and the subarachnoid space had not been blocked completely, postoperative MRI study showing intrathoracic meningocele still existed. In addition, as laminectomy had been performed but the dural membrane had not been sutured by watertight, the patient had extradural effusion and spinal deformity after the operation. Thus, the most appropriate strategy is to disconnect the cyst from the CSF and subarachnoid space followed by the removal of the arachnoid cyst in the spinal canal. Furthermore, laminoplasty could better prevent spinal deformation.^[23]^

## 5 Conclusion

The presented 4 cases are remarkable with many considerable much experiences of both success and failure. Anterior spinal meningocele is a kind of rarely encountered malformation with the unclarified mechanism of pathogenesis. Imaging is critical, but a detailed medical history carefully taken by medical service providers can prompt important clues. Conservative management is an appropriate option for small, asymptomatic anterior spinal meningocele. The key to surgery is to disconnect the cyst from the CSF and subarachnoid space. Multiple approaches are available, but each approach taken into account must be carefully judged and weighed. The decision should be made on the basis of the specific surgical goals and individualized management should be applied to different cases.

## Author contributions

**Data curation:** Jiagang Liu, SiQing Huang.

**Funding acquisition:** Jiagang Liu.

**Investigation:** Jiagang Liu.

**Resources:** Jiagang Liu.

**Supervision:** JianGuo Xu.

**Writing – original draft:** YueLong Wang.

**Writing – review & editing:** YueLong Wang, Bin He, Jinhao Yang, JianGuo Xu, SiQing Huang.
